# Genome-Wide Identification and Characterization of the Mitochondrial Transcription Termination Factors (mTERFs) in *Capsicum annuum* L.

**DOI:** 10.3390/ijms21010269

**Published:** 2019-12-31

**Authors:** Bingqian Tang, Lingling Xie, Ting Yi, Junheng Lv, Huiping Yang, Xiang Cheng, Feng Liu, Xuexiao Zou

**Affiliations:** 1 Longping Branch, Graduate School of Hunan University, Changsha 410125, China; bqtang@126.com (B.T.); maydayfdt10@163.com (T.Y.); junhenglv@hnu.edu.cn (J.L.); 18375723982@163.com (H.Y.); cheng13548763217@163.com (X.C.); 2 Hunan Vegetable Research Institute, Changsha 410125, China; xllxie@sina.cn; 3 College of Horticulture and Landscape, Hunan Agricultural University, Changsha 410125, China

**Keywords:** capsicum, mTERF gene family, phylogenetic analysis, expression analysis, GO analysis, abiotic stress

## Abstract

Mitochondrial transcription termination factors (*mTERFs*) regulate the expression of mitochondrial genes and are closely related to the function of the mitochondrion and chloroplast. In this study, the *mTERF* gene family in capsicum (*Capsicum annuum* L.) was identified and characterized through genomic and bioinformatic analyses. Capsicum was found to possess at least 35 *mTERF* genes (*CamTERFs*), which were divided into eight major groups following phylogenetic analysis. Analysis of *CamTERF* promoters revealed the presence of many cis-elements related to the regulation of cellular respiration and photosynthesis. In addition, *CamTERF* promoters contained cis-elements related to phytohormone regulation and stress responses. Differentially expressed genes in different tissues and developmental phases were identified using RNA-seq data, which revealed that *CamTERFs* exhibit various expression and co-expression patterns. Gene ontology (GO) annotations associated *CamTERFs* primarily with mitochondrion and chloroplast function and composition. These results contribute towards understanding the role of *mTERFs* in capsicum growth, development, and stress responses. Moreover, our data assist in the identification of *CamTERFs* with important functions, which opens avenues for future studies.

## 1. Introduction

Most of the genes within mitochondria and chloroplasts have been lost or transferred to the nucleus during the evolution of bacterial progenitors of different plant species; however, many important genes remain in mitochondrion and chloroplast genomes to coordinate processes such as gene expression, photosynthesis, and the electron transport chain [1,2]. Although mitochondrion and chloroplast genes retain the characteristics of prokaryotic expression, they have expanded and evolved with nuclear genes in plants, resulting in molecular mechanisms of transcription and replication that are more complicated than those of their progenitors [3]. Mitochondrial transcription termination factor (mTERF) proteins belong to a protein superfamily characterized by an mTERF motif (a leucine zipper consisting of 30 leucine residues). mTERFs are encoded by nuclear genes, which target the mitochondria following translation [4]. mTERFs are involved in the regulation of mitochondrial DNA replication as well as gene transcription and translation through their binding to mitochondrial nucleic acid [5].

mTERFs were first characterized in animal mitochondria and denoted transcription termination factors. mTERFs are grouped into four subfamilies, with different subfamilies having different biological functions according to the number and location of mTERF motifs [5,6]. In metazoans, mTERFs are important transcription termination factors [7,8]. In mice, mTERF3 and mTERF4 are required for embryogenesis—mTERF3 binds to the promoter region of mitochondrial DNA, initiating negative transcription regulation [9], whereas mTERF4 controls the biogenesis and activity of mitochondrial ribosomes by recruiting rRNA large subunit methyltransferase [10]. In humans, mTERF3 and mTERF4 function through binding RNA. In addition, in vitro experiments showed that mitochondrial DNA mediates the interactions between different mTERF proteins, indicating that mTERF functions are differentiated by the mitochondrial DNA properties of different organisms [11].

In plants, the expression of mitochondrial genes is essential for plant development, photosynthesis, and cellular respiration. Mitochondrial gene expression requires the specific binding of various nuclear-encoded proteins to mitochondrial DNA (mtDNA) to facilitate RNA transcription, splicing, modification, and regulation of the translation process. MOCs are the mTERF-like proteins of *Chlamydomonas reinhardtii*. MOCs comprise six subfamilies, namely MOC1–6, which have a similar function to that of SOLDAT10 in *Arabidopsis* (*Arabidopsis thaliana*) [12]. Past research reported that MOC1 function was related to hydrogen generation during the photoreaction process [13], and MOC1 loss-of-function renders *Chlamydomonas* sensitive to strong light, destroys the stability of the mitochondrial respiratory chain complex, and also results in the termination of mtDNA transcription [14].

Compared with bryophytes or algae, there is a greater abundance of *mTERF* genes in angiosperm genomes, which encode proteins localized to mitochondria or chloroplasts that function in plant development and stress resistance [15]. Queries of PlnTFDB, a transcription factor database (http://plntfdb.bio.uni-potsdam.de/v3.0/) [16], shows that *Arabidopsis* has 35 *mTERFs* [17], rice (*Oryza sativa* subsp. Japonica) has 30 *mTERFs*, and maize (*Zea mays*) has 31 *mTERFs* [18].

Research on the function of mTERFs and their mechanisms is currently limited. Several studies on *mTERFs* in *Arabidopsis* were reported. *mTERF1/2/5/6/8/9/10/11/12* were shown to localize to chloroplasts, with *mTERF6* also targeting the mitochondria [17,19]. *soldat10/flu* double mutant seedlings affected in mTERF1 in the *flu* mutant background did not display the lethal ^1^O_2_ generation upon a dark-to-light shift (typical of the *flu* mutation because of disrupted chloroplast homeostasis), indicating a role for *mTERF1* in plastid signaling and chloroplast biogenesis [12]. The cotyledons, leaves, stems, and bracts of the *mTERF5* deletion mutant *mda1* are lighter in color, and *mda1* plants are more tolerant of osmotic stress and less ABA(Abscisic acid) sensitive [20]. PED191 (*mTERF6*) functions in the development of leaf color, with early chloroplast development blocked in the corresponding mutant, resulting in albino seedlings or lethality [21]. Past research has shown that the nuclear-encoded mTERF6 protein binds to an RNA sequence located in the tRNA for Ile(GAU), and a relative deficit in mature chloroplast ribosomal RNAs in *mterf6* mutants leads to a reduction in the number of functional ribosomes, thus compromising chloroplast protein synthesis and consequently perturbing chloroplast development and plant growth [22]. Recently, it was shown that mTERF6 directly associates with the 3′-end sequence of the rpoA polycistron in vitro and in vivo, and that the lack of mTERF6 promotes read-through transcription at this site, thus further demonstrating the essential role of mTERF6 in chloroplast gene expression and development [23]. Through characterization of the *mterf6-5* mutant, it was found that the corresponding mutation enhanced the leaf phenotype of the asymmetric leaf mutant *as1-1*, causing leaves to appear radial, and led to lethality in the early stage of vegetative development, revealing a further role for mTERF6 in leaf patterning and highlighting the importance of mTERFs in plant development [24]. PTAC15 (*mTERF8*) was purified from an *Arabidopsis* transcriptionally active plastid chromosome complex and proposed to take part in the transcriptional regulation of chloroplast DNA [25]. Recent RNA blot analysis showed that a larger transcript derived from the psbEFLJ polycistron accumulates in the *mterf8* mutant than that observed in the wild type, and electrophoresis mobility shift assays, chloroplast chromatin immunoprecipitation, and transcriptional analysis using the in vitro T7 RNA polymerase system showed that mTERF8 specifically binds to the 3′terminal region of psbJ, suggesting that mTERF8 is specifically involved in the transcription termination of the chloroplast gene psbJ [26]. In *Arabidopsis*, RUG2/BSM (*mTERF4*) is localized to chloroplasts and mitochondria, and its loss of function leads to dysplasia of chloroplasts and mitochondria, homozygous embryonic developmental stagnation, as well as altered gene expression in chloroplasts, mitochondria, and the nucleus, suggesting that it may be a key factor meditating communication between chloroplasts/mitochondria and the nucleus [27]. In maize, *Zm-mTERF4* coimmunoprecipitates with many chloroplast introns and the splicing of some of these introns is disrupted even in hypomorphic *Zm-mterf4* mutants. Furthermore, the splicing of two transfer RNAs (trnI-GAU and trnA-UGC) and one ribosomal protein messenger RNA (rpl2) is particularly sensitive to the loss of Zm-mTERF4, accounting for the loss of plastid ribosomes in *Zm-mterf4* mutants. These findings extend the known functional repertoire of the mTERF family to include group-II intron splicing and suggest that a conserved role for mTERFs in chloroplast RNA splicing underlies the physiological defects associated with mutations in *RUG2*/*BSM*, the *Zm-mTERF4* ortholog in *Arabidopsis* [28]. An analysis of the effects of *mTERF9* mutation revealed functions for mTERF9 in ABA-, salt-, and osmotic-stress responses [29]. *mTERF11* over-expression plants displayed enhanced salt tolerance when grown on MS media with added NaCl [19]. Moreover, the over-expression of *mTERF10* promoted seed germination and seedling growth on NaCl-containing MS media with added ABA, indicating that *mTERF10* may play a role in ABA-mediated salt tolerance [19].

The above-mentioned studies indicate that the majority of chloroplast-associated *mTERFs* regulate the function of chloroplasts by regulating the expression of chloroplast and nuclear genes, which in turn affects leaf morphology and color and some abiotic-stress responses. Moreover, mTERF15 and mTERF18 are specifically localized to mitochondria [30]. The *mTERF18* mutant (*shot1*) is characterized by dark green leaves, low ROS accumulation, high heat resistance, and the upregulation of both respiratory electron transport chain-related and various stress-response genes [31]. *mTERF15* functions in the plant growth cycle [30]. Mitochondria-associated *mTERFs* may play roles in the stability of mitochondrial gene expression and mitochondrial function. 

The capsicum (*Capsicum annuum* L.) complete genome sequence [32,33] has laid the foundation for further study of the functions of capsicum genes and the relationships between them. However, characterization of the capsicum *mTERF* (*CamTERF*) family has not been reported yet. Here, we identified *CamTERFs* through bioinformatic analysis, then characterized their basic properties, chromosomal locations, tandem repeats, evolutionary relationships, and promoter cis-elements, as well as their tissue, organ, fruit-development, and abiotic-stress expression patterns. This study aimed to provide a foundation for the understanding of *CamTERF* functions in normal plant developmental processes and under osmotic stress.

## 2. Results

### 2.1. Basic Characterization of CamTERFs and Comparison between Available Capsicum Genome Databases

Based on bioinformatic methods, a total of 35 and 34 *CamTERFs* were identified in the Zunla-1 and CM334 genome databases, respectively. These candidates were further refined through SMART conserved motif and iTAK transcription factor identification. The two groups of candidate *CamTERFs* were subjected to a blastp alignment in order to identify those homologous genes whose shared sequence identity was more than 95%. 

The 35 Zunla-1 *CamTERFs* were categorized by their chromosomal locations, and denoted *CamTERF1–35*. These Zunla-1 *CamTERFs* were primarily used for the remainder of the analyses in this study. The longest *CamTERF* was *CamTERF29*, comprising 1971 amino acid residues, whereas the shortest *CamTERF* was *CamTERF20*, comprising only 327 residues (Table 1). In addition, no homologous gene in the CM334 group was identified for *CamTERF20*. The isoelectric points of *CamTERFs* ranged from 6.1 (*CamTERF28*) to 10.02 (*CamTERF22* and *CamTERF27*), and their molecular masses ranged from 12.4 KD (*CamTERF20*) to 67.82 KD (*CamTERF10*). 

Considering the incompleteness of the capsicum genome, those genes whose chromosomal information was unclear were denoted as “00g”. There were 34 homologous gene pairs identified between Zunla-1 and CM334, of which 19 pairs exhibited the same chromosomal annotation information. Ten genes (*CamTERF5*, *CamTERF15*, *CamTERF16*, *CamTERF17*, *CamTERF18*, *CamTERF21*, *CamTERF24*, *CamTERF25*, *CamTERF29*, and *CamTERF33*) that had chromosomal records in the Zunla-1 genome had no annotation in the CM334 genome; seven of these genes were located on Chr4 according to the Zunla-1 genome. Two genes (*CamTERF2* and *CamTERF3*) that had chromosomal information in the CM334 genome had no chromosomal annotation in the Zunla-1 genome. Three genes (*CamTERF19*, *CamTERF23*, and *CamTERF34*) were annotated as localized on different chromosomes in either genome database. These results indicate that both the Zunla-1 and CM334 databases cannot provide complete capsicum genome annotation information. For *CamTERFs*, the most remarkable difference was observed on chromosome 4. Further studies on *CamTERFs* would contribute to improved annotation of the two genome databases.

### 2.2. Analysis of CamTERFs Chromosomal Location and Subcellular Localization

**Figure 1:**
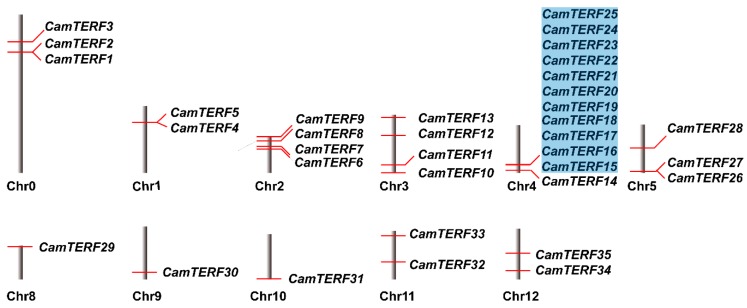
Chromosomal location and gene duplication of capsicum *mTERFs* (*CamTERFs*). The tandem duplicated genes are marked by blue rectangles.

**Figure 2:**
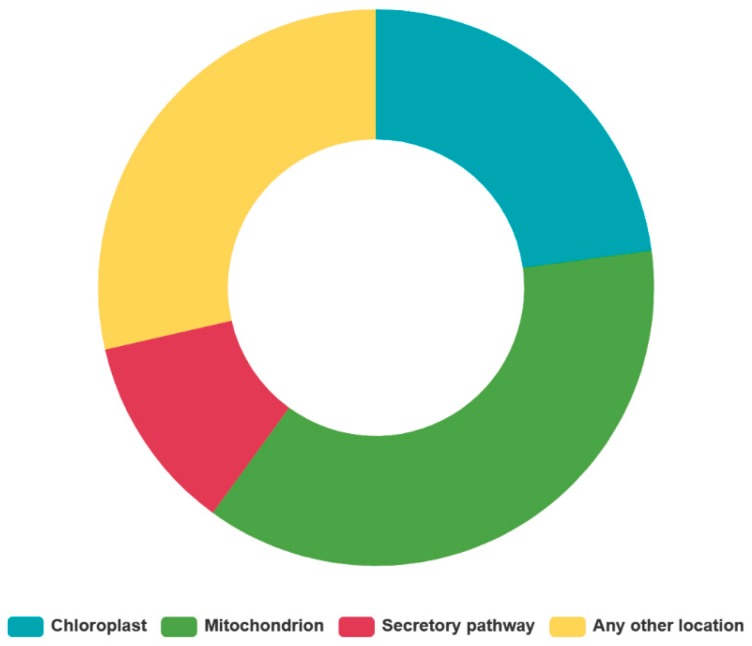
CamTERFs protein predicted of the Subcellular localization.

Figure 1 depicts the distribution of the 35 *CamTERFs* across the ten capsicum chromosomes. Those genes whose chromosomal location was unavailable are denoted here as Chr0. Analysis of the chromosomal location of *CamTERFs* illustrated that there are no *CamTERFs* on Chr6 and Chr7, and that Chr4 contained the most *CamTERFs* (12 genes). Among these 12 *CamTERFs*, 11 are tandem repeat genes that are symmetrically distributed at the front end of chromosome 4, with a length of 36.52–37.05 Mb. Chr2, Chr3, and Chr5 were found to contain three to four *CamTERFs*, whereas Chr1, Chr8, Chr9, Chr10, and Chr11 contain only one to two *CamTERFs*. In general, *CamTERFs* showed a cluster phenomenon in specific segments of chromosome 4, whereas they were relatively scattered across the other chromosomes. This arrangement may be related to the tandem repeat event on Chr4. Table 2 and Figure 2 describe how most *CamTERFs* were found to be localized in mitochondria or chloroplasts, thus leading to the presumption that the functions of *CamTERFs* are related to these organelles. Cellular localization prediction based on protein sequence indicated that there are 14 CamTERFs not associated with either chloroplasts or mitochondria. These proteins are of interest and further experimental verification is needed to determine their cellular localization as part of the continual functional research of CamTERFs.

### 2.3. Phylogenetic Relationships, Gene Structures, and Motifs of CamTERFs

**Figure 3:**
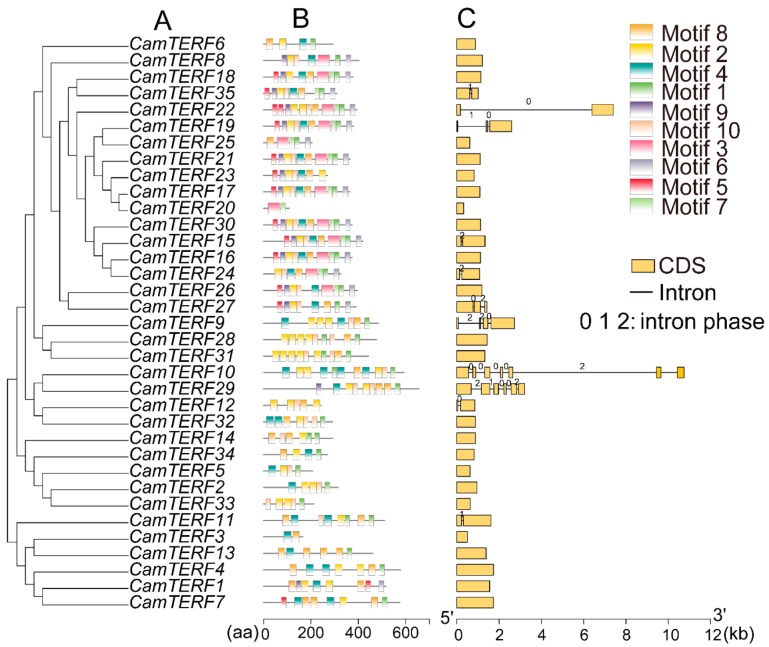
Phylogenetic relationship, conserved motif, and gene structure analysis of *CamTERFs*. (**A**) Phylogenetic tree of 35 *CamTERFs*. Depicted is an unrooted neighbor-joining phylogenetic tree; (**B**) Distributions of conserved motifs in *CamTERFs*. Ten putative motifs are indicated with different colored boxes. For details of motifs refer to Table 2; (**C**) Exon/intron organization in *CamTERFs*. Yellow boxes represent exons and black lines represent introns. The numbers 0, 1, and 2 represent the intron splicing phase. Exon length can be inferred by the scale at the bottom.

Figure 3 depicts the structure of each *CamTERF*, including the number of introns and exons and intron phase, which may be related to evolution and gene function. Intron phase among *CamTERFs* was found to vary from phase 0 to phase 2. For phase 0 introns, the exons connected on both sides can be rearranged and connected without limits. Results of the gene structure analysis showed that 24 *CamTERFs* (68.6% of the entire group of 35 *CamTERFs*) have no introns, 6 *CamTERFs* (17.1%) have only one intron, and 5 *CamTERFs* (14.3%) have more than one intron. *CamTERF10* was found to have six introns, which represents the greatest number of introns among all *CamTERFs*. In addition, five of the introns in *CamTERF10* are phase 0. Furthermore, ten conserved motifs in *CamTERFs* were predicted using the MEME(Multiple Em for Motif Elicitation) website, which were found to consist of 21–50 amino acids (sequences are shown in Table 3).

### 2.4. Homologous Relationships among mTERFs of Capsicum, Rice, and Arabidopsis

**Figure 4:**
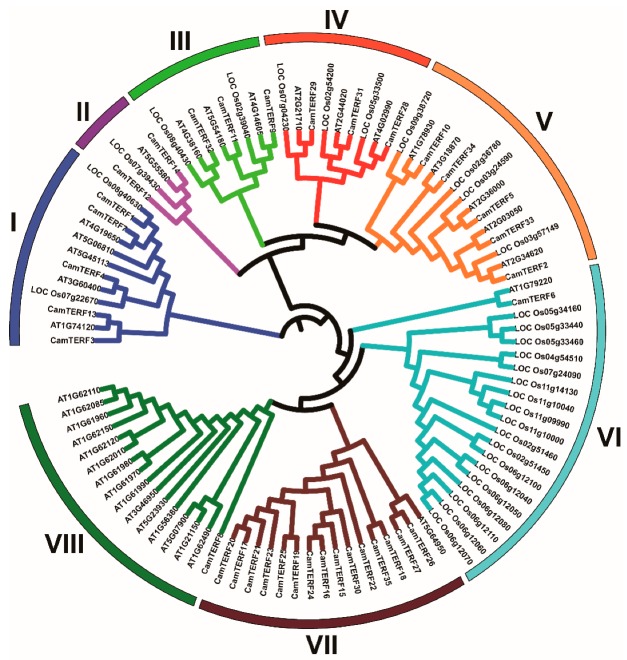
Phylogenetic tree of *mTERFs* from *Arabidopsis*, rice, and capsicum. The phylogenetic tree was constructed using the NJ (neighbor-joining) method with 1000 bootstrap replications. The eight subfamilies are distinguished with different colors.

In order to better understand the homologous evolutionary relationship among *mTERFs* of capsicum and the model species rice and *Arabidopsis*, the phylogenetic relationships between *mTERFs* of capsicum, *Arabidopsis*, and rice were analyzed by generating a phylogenetic tree constructed using the adjacency algorithm (NJ). The resulting phylogenetic tree consists of 30 *mTERF* sequences from rice, 34 *mTERF* sequences from *Arabidopsis*, and 35 *mTERF* sequences from capsicum, as shown in Figure 4. According to the gene family classification of *Arabidopsis* [17] and the *mTERF* phylogenetic tree, *mTERFs* of the three analyzed species were classified into eight groups. Group VI and VIII contained the least members, with only one *CamTERF* included in each. Group VII had the largest number of members, including 15 *CamTERFs*. Furthermore, group II had two *CamTERFs*, groups III and IV had three *CamTERFs*, and groups I and V had five *CamTERFs*. 

Phylogenetic analysis showed that groups VI, VII, and VIII were mainly enriched in the *mTERFs* of rice, capsicum, and *Arabidopsis*, respectively. The remaining *mTERFs* of the three species were distributed fairly evenly among groups I to V. These results suggest that groups VI, VII, and VIII represent specific gene clusters of rice, capsicum, and *Arabidopsis*, respectively, which were formed during evolution of the *mTERF* family.

### 2.5. Analysis of CamTERF Promoter cis-Elements

**Figure 5:**
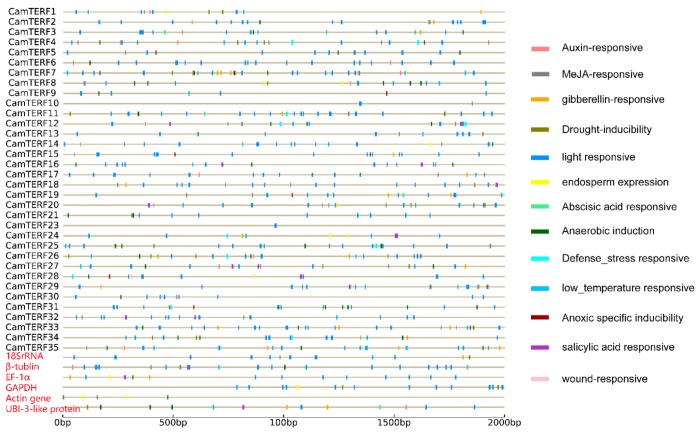
Predicted cis-elements in *CamTERFs* promoters. Promoter sequences (−2000 bp of translation start site) of 35 *CamTERFs* (promoter sequence of *CamTERF22* was omitted) and 6 housekeeping genes were analyzed by PlantCARE.

To further investigate the potential regulatory mechanisms of *CamTERFs* in chloroplast and mitochondrion functions and abiotic-stress responses, the 2 kb sequence upstream of the translation initiation site of each *CamTERF* was tested using the PlantCARE tool, and also six housekeeping genes were chosen in pepper [34]—specific information about those genes are shown in Table S1. Potential *CamTERF* cis-elements were obtained, as shown in Figure 5, and the housekeeping genes information are in red. The *CamTERFs* promoter sequences were found to contain many abiotic stress-related elements, such as light-responsive elements, many hormone regulation-related cis-elements, such as those responsive to auxin, MeJA(Methyl Jasmonate), gibberellin, abscisic acid, anoxic conditions, and salicylic acid, as well as many osmotic stress-related cis-elements, such as those responsive to low temperature and drought. Among the six housekeeping genes, *18SrRNA*, *β-tublin*, *EF-1a*, and *GAPDH* have many light responsive elements, however *Actin gene* and *UBI-3-like protein* contain less. Compared with six housekeeping genes, *CamTERF2*,*6*,*7*,*11*,*14* are more light-responsive and *CamTERF7* is more gibberellin-responsive.

### 2.6. Analysis of CamTERFs Expression Patterns and GO Enrichment Analysis of CamTERFs Co-Expressing Genes

**Figure 6:**
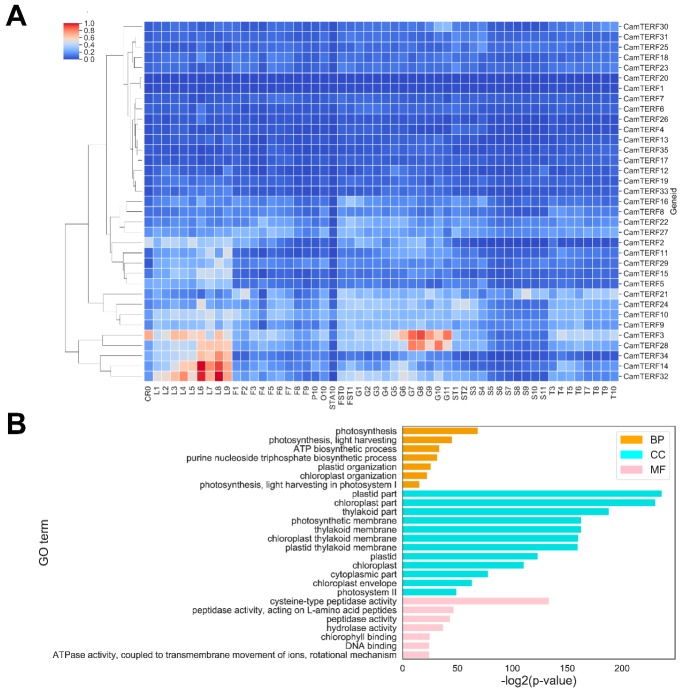
(**A**) Expression profiles of *CamTERFs* in different tissues and organs. CR0: root control; L1–L9: Leaf from 0 DAG to 50 DAG; F1–F9: Flower from 0 DAG to 50 DAG; P10: Petal; O10: Ovary; STA10: Stamen; FST0 and FST1: Whole fruit from 3 DAP and 7 DAP; G1–G11: Pericarp from 10 DAP to 60 DAP; ST1 and ST2: Placenta and Seed from 10 DAP to 15 DAP; S3–S11: Seed from 20 DAP to 60 DAP; T3–T10: Placenta from 20 DAP to 60 DAP. DAG: Days after germination; DAP: Days after pollination; (**B**) Analysis of gene ontology (GO) enrichment for co-expressing *CamTERFs*. GO term categorization includes biological process (BP), cellular component (CC), and molecular function (MF).

A rich set of tissue expression data was obtained by RNA-seq analysis using different tissues in different developmental phases alongside the *CamTERFs* RNA-seq FPKM (Fragments Per Kilobase Million) data list detailed in Table S2 and the sample data list detailed in Table S3. The expression patterns of *CamTERFs* in different tissues were illustrated with a heat map (Figure 6A). The horizontal axis shows the names of different tissues in different developmental phases and treatment conditions. Except for *CamTERF1* and *CamTERF20*, expression of the majority of *CamTERFs* was detected in at least one tissue type. *CamTERF3*, *CamTERF28*, *CamTERF34*, *CamTERF14*, and *CamTERF32* were found to be expressed in all tissues. These genes displayed their highest expression level in leaves and peels, lowest expression level in seeds, and a moderate expression level in other tissues. Moreover, these five genes exhibited significant differential expression over the nine developmental phases of leaf tissue. With progressing leaf development, the expression levels of these five genes first increased and then decreased, suggesting that they function in the regulation of leaf development. 

GO (Gene ontology) enrichment analysis was used to understand the function of individual and co-expressed *CamTERFs*. As depicted in Figure 6B, the resulting associated terms were classified into biological process (BP), molecular function (MF), and cellular component (CC). Highly-enriched GO terms included photosynthesis (GO:0015979), photosynthesis, light harvesting (GO:0009765), and ATP biosynthetic process’ (GO:0006754) in the BP category; plastid part (GO:0044435), chloroplast part (GO:0044434), and photosynthetic membrane (GO:0034357) in the CC category; and cysteine-type peptidase activity (GO:0008234), peptidase activity, acting on l-amino acid peptides (GO:0070011), and peptidase activity (GO:0008233) in the MF category. The co-expressed *CamTERFs* were associated with 222 GO terms, as listed in Table S4.

### 2.7. Analysis of the Expression of CamTERFs under Salt Stress and ABA Treatment

**Figure 7:**
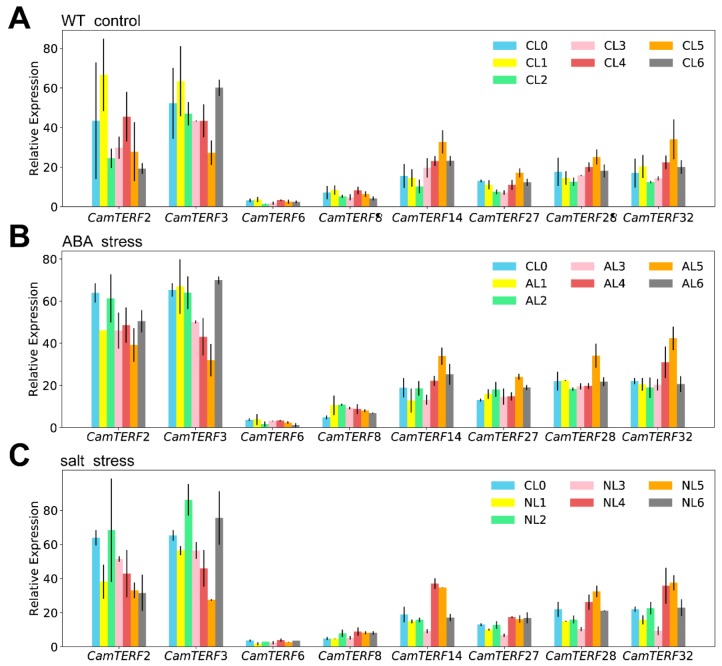
(**A**) Expression profiles of *CamTERFs* untreated following a 24 h time course; (**B**) expression profiles of *CamTERFs* under ABA (Abscisic acid) stress following a 24 h time course; (**C**) expression profiles of *CamTERFs* under salt stress and following a 24 h time course.

To further explore the expression changes of *CamTERFs* under abiotic stresses such as salt and ABA treatment, several candidate genes were first selected for analysis. According to the phylogenetic tree (Figure 4), mTERFs of capsicum, rice, and *Arabidopsis* were classified into eight subclasses. A representative *CamTERF* was selected from each of these eight subclasses. Experimental leaf samples were collected from plants treated with NaCl or ABA and relative gene expression levels of the eight candidate genes were determined by RT-qPCR (Reverse transcription quantitative PCR) (Figure 7). Samples used the untreated leaf at each timepoint as control to eliminate the effects of circadian rhythm on gene expression changes.

The relative expression of *CamTERF2* decreased after 1 h of ABA and salt treatment compared with untreated (Figure 7A), and then increased with some fluctuation in expression level. The expression of *CamTERF3* showed a similar trend to that of *CamTERF2* at the early stage of treatment; the relative expression levels of *CamTERF14*, *CamTERF27*, *CamTERF28*, and *CamTERF32* increased after 1–12 h of ABA treatment, followed by decreased expression by 24 h after treatment, they showed a similar trend to the untreated control group. The relative expression levels of *CamTERF6* and *CamTERF8* changed only slightly, without drastic fluctuation. These two genes also exhibited low expression levels in different tissues (Figure 6A), suggesting that ABA treatment has little impact on *CamTERF6* and *CamTERF8*. The expression levels of the eight candidate genes in those plants treated with NaCl exhibited similar patterns to those in ABA-treated plants.

## 3. Discussion

Whole-genome sequencing of capsicum was completed in 2014 [32,33]. However, annotation of the capsicum genome has been difficult because it contains many repeat sequences. Thus far, the annotation file for capsicum remains not comprehensive. According to the published data, 34,903 genes of the CM334 capsicum genome have been annotated, and 9938 of these have not been localized to any particular chromosome; *CamTERFs* falling into this category were denoted here as Chr00. CM334 is a native cultivar of Mexico and Zunla is a cultivar of Guizhou, China. Both cultivars belong to *Capsicum annuum*; however, there are some geographical and environmental differences between habitats of *Capsicum annuum*, so genomic differences between different cultivars are normal. A comparison of the CM334 and Zunla-1 capsicum genomes has the potential to improve current annotation information. 

In this study, 35 and 34 *CamTERFs* were separately identified in the Zunla-1 and CM334 capsicum genomes, respectively, based on bioinformatics methods. The homologous genes in these two genomes were identified through BLASTP (Basic Local Alignment Search Tool Protein) alignment. Among the 34 pairs of homologous genes, *CamTERF4* is located on Chr1, *CamTERF6/7/8/9* are located on Chr2, *CamTERF10/11/12/13* are located on Chr3, *CamTERF14/22* are located on Chr4, *CamTERF26/27/28* are located on Chr5, and *CamTERF35* is located on Chr12. *CamTERF2/3* (Capana00g003424 and Capana00g003479) were assigned ‘00g’ in the Zunla-1 genome, whereas their homologs in the CM334 genome (CA01g01340 and CA04g12790) are located on Chr1 and Chr4, respectively. Thus, the CM334 annotation would help to improve the Zunla-1 genome. In addition, those genes with no chromosomal location specified in the CM334 genome, namely *CA00g83430*, *CA00g83420*, *CA00g83410*, *CA00g83400*, and *CA00g85840*, were annotated as located on Chr4 in the Zunla-1 genome. These two genomes can therefore complement each other. Furthermore, the chromosomal location map showed that *CamTERFs* are distributed unevenly on Chr10 and no *CamTERFs* are present on Chr6 and Chr7. Twelve *CamTERFs* located at the front of Chr4 form a gene cluster, where a tandem repeat has been detected through blast analysis. 

In order to study the gene evolution and transcriptional features of *CamTERFs*, we analyzed their gene structures in detail, specifically the number and distribution of introns and exons. The structure of *CamTERFs* is simple, with 0–6 introns. Genes within the same subclass have similar structures and predicted motifs. These results indicate that the gene structure of *CamTERFs* is highly conserved. 

To study the evolutionary relationships between *mTERFs* of capsicum, rice, and *Arabidopsis*, a phylogenetic tree was constructed through cluster analysis. By combining this phylogenetic tree with *mTERFs* classification in rice and *Arabidopsis*, *mTERFs* among the compared species were divided into eight subclasses. As a result of highly conserved features, those *mTERFs* within the same subclass showed similar functions. The functions of many *mTERFs* in *Arabidopsis* have been verified. For example, *mTERF5/9/10/11* play roles in the resistance to salt and osmotic stress, and *mTERF5/9/10* also function in ABA regulation [19,20]. *mTERF1/6* are involved in chloroplast biogenesis and regulate leaf color [12,21], which are processes dependent on their chloroplast localization. *mTERF15/18* are located in the mitochondria [30]. The *shot1* mutant phenotype, caused by *mTERF18* loss-of-function, is characterized by a dark green leaf color, decreased ROS accumulation, high heat resistance, and the upregulation of many mitochondrial respiratory electron transport chain-related and various stress-response genes [31]. *mTERF15* is involved in the plant growth cycle [30]. Finally, mitochondria-associated *mTERFs* may be closely related to the stability of mitochondrial gene expression or mitochondrial function. 

Promoter analysis of *CamTERFs* identified cis-elements in *CamTERF2*,*6*,*7*,*11*,*14*, suggesting that the expression of *CamTERFs* might be regulated upon external stimuli, such as light. However, as the promoter analysis is based on prediction, it requires further experimental verification. Functional studies of mTERFs in *Arabidopsis* could provide the possibility for the characterization of *CamTERFs*, but the functions of *CamTERFs* homologs needs further experimental verification.

*CamTERFs* expression patterns in 11 different plant tissues, including roots, leaves, flowers, fruits, seeds, petals, ovary, anther, and placenta, were investigated (Figure 6A). This analysis showed that the expression of *CamTERFs* is tissue-specific during plant development. *CamTERFs* exhibit the highest expression in leaves and peels, in which *CamTERF3/28/34/14/32* are expressed relatively higher and present remarkable tissue-specific expression differences. By contrast, *CamTERF1* and *CamTERF20* have almost no detectable expression in different tissues and organs.

In GO enrichment analysis, terms associated with co-expressed genes were highly enriched for plastid part (GO:0044435) and chloroplast part (GO:0044434) in the CC category (Figure 6B). A large number of co-expressed genes were classified into mitochondria- and chloroplast-related biological processes, such as photosynthesis (GO:0015979), photosynthesis, light harvesting (GO:0009765), and ATP biosynthetic process (GO:0006754). Combined with the identified *CamTERFs* promoter cis-elements, GO analysis results suggest that *CamTERFs* are closely tied to plant respiration and photosynthetic processes, in which chloroplasts and mitochondria participate. In agreement, a recent study in *Arabidopsis* has shown that mTERF5 acts as a transcriptional pausing factor to positively regulate the transcription of chloroplast *psbEFLJ* [35].

Plant mTERFs can regulate the expression of stress resistance-related genes and result in improved plant stress resistance, as has been demonstrated for the regulation of ABA signaling and salt tolerance [19,20]. In this study, RT-qPCR analysis showed that the expression of some *CamTERFs* was considerably upregulated under ABA and salt stress. Of note, *CamTERF2* and *CamTERF3* exhibited altered expression patterns following ABA and NaCl treatment; their expression levels increased initially, then decreased, and then increased again during late stages of the time course following experimental treatment. *CamTERF2* shares high sequence similarity with its homolog *mTERF10* in *Arabidopsis* (E: 10^−100^). Past research suggested that mTERF10 participates in plant salt resistance through an ABA-meditated mechanism [19] and *CamTERF3* shares high sequence similarity with its homolog *mTERF12* in *Arabidopsis* (E: 3 × 10^−52^). *mTERF10/11/12* in *Arabidopsis thaliana* belong to the same subclass and are mitochondria-related nuclear genes that encode chloroplast proteins. It has been reported that *mTERF10/11* are involved in salt-stress responses in *Arabidopsis*; however, no evidence supports a similar role for *mTERF12* [19]. For *CamTERF2* and *CamTERF3* in capsicum, we observed that their expression levels are relatively active and responsive to ABA and salt stress, but further investigation is necessary to prove that they participate in ABA- and salt-stress responses in capsicum.

## 4. Materials and Methods 

### 4.1. Capsicum mTERF Family Identification

*Capsicum annuum* L. protein data at the whole-genome level were downloaded from two databases, pepper CM334 (http://peppergenome.snu.ac.kr/download.php) and Zunla-1 (ftp://ftp.ncbi.nlm.nih.gov/genomes/refseq/plant/Capsicum_annuum/latest_assembly_versions/GCF_000710875.1_Pepper_Zunla_1_Ref_v1.0). Protein sequences of the *mTERF* family of *Arabidopsis* (*Arabidopsis thaliana*) were obtained from the *Arabidopsis* genome database (https://www.arabidopsis.org/), which were used as query sequences to be aligned with the two pepper protein files. Thirty candidate *CamTERF* family members were identified in CM334 and 32 candidates were identified in Zunla-1. 

Next, the Pfam ID of mTERF (PF02536) [17,36] was retrieved from the Pfam website (http://pfam.janelia.org/) [37]. In Pfam, the file named mTERF.hmm was downloaded, which contained the conserved domain information of the *mTERF* family. Similar domains between the two capsicum datasets were matched by using hmmer software’s hidden Markov algorithm [38] with a parameter of e-value ≤ 1 × 10^−5^. As a result, 34 candidate *CamTERFs* were identified in CM334 and 35 candidates were identified in Zunla-1.

The candidate members of the *CamTERF* family obtained from the two databases were used as inputs in SMART analysis (http://smart.embl-heidelberg.de/#opennewwindow) [39] to identify whether they possessed the conserved *mTERF* domain. The database of iTAK (http://itak.feilab.net/cgi-bin/itak/index.cgi) defined the transcription factor (TF) family and relative rules. By identifying the TFs [16,40,41,42] through hmmscan, true *CamTERF* members were finally obtained. 

### 4.2. Basic Characterization and Homology of CamTERFs

Biochemical properties of CamTERFs, such as isoelectric point, molecular weight, and length, were analyzed using the online tool ExPASy (http://web.expasy.org/protparam/) [43]. CamTERF sequences obtained from the CM334 database were aligned with the sequences from Zunla-1 database, after which the most similar CamTERFs between these two databases were set aside. 

### 4.3. Analysis of Chromosomal Location and Subcellular Localization

Python script was used to extract the position and length information of each gene from the gff and fasta files of the *CamTERFs*, and also extract the position and length of the *CamTERFs* on each chromosome. *CamTERFs* chromosomal positioning was visualized using python packages, i.e., pandas, numpy, and matplotlib, alongside the indication of tandem repeat genes. The subcellular localizations of CamTERFs were predicted using CamTERF protein sequences in the TargetP-2.0 Server (http://www.cbs.dtu.dk/services/TargetP). TargetP 2.0 is a novel state-of-the-art method used to identify N-terminal sorting signals, which direct proteins to the secretory pathway, mitochondria, and chloroplasts or other plastids. This method works by examining the strongest signals from the attention layer in the network and identifying whether the second residue in the protein, that is, the one following the initial methionine, has a strong influence on the classification [44].

### 4.4. Analysis of Gene Structure, Phylogeny, and Conserved Motifs

The online website MEME (http://meme-suite.org/) was used to predict the conserved motifs within *CamTERFs*. Based on protein evolution tree files and annotation files, the evolutionary relationships, gene structures, and predicted motifs of *CamTERFs* were illustrated using TBtools [45].

### 4.5. Analysis of Homologous Evolutionary Relationships between mTERFs of Rice, Arabidopsis, and Capsicum

Multiple sequence alignment of capsicum, rice (*Oryza sativa*), and *Arabidopsis mTERFs* was conducted using MUSCLE (Multiple Protein Sequence Alignment) under Linux. Contiguous algorithm was used to build phylogenetic tree files through treebest software [46] and a phylogenetic tree was illustrated by using the online tool iTOL (Interactive Tree Of Life; https://itol.embl.de/) [39].

### 4.6. Analysis of Cis-Regulatory Elements in CamTERFs

For *CamTERFs*, the nucleotide sequences 2 kb upstream of the translation start site of each gene were uploaded into the PlantCARE tool (http://bioinformatics.psb.ugent.be/webtools/plantcare/html/) [47]. Then, the cis-elements within each promoter sequence were predicted, which were used to identify the regulatory elements that related to *CamTERFs*. Results were visualized by the python matplotlib package.

### 4.7. Analysis of CamTERFs Expression and GO Enrichment for CamTERF Co-Expressing Members

A capsicum variety named 6421, which was identified in our lab, was used in this research (this line was supplied by the Vegetable Research Institute of Hunan Academy of Agricultural Sciences). Tissues representing 11 plant organs, including leaves, flowers, peels, placenta, seeds, and roots, were sampled during different developmental phases. Then, these samples were analyzed by RNA-Seq. The RNA-Seq data were visualized on the PepperHub [48]. Gene expression data in different tissue and during different phases were analyzed by using HISAT2, featureCounts [49,50,51]. The FPKM value was calculated and normalized by using the MAX/MIN method. Cluster analysis was performed by the UPGMA (unweighted pair-group method with arithmetic means) method and visualized with heat map, which was generated with Python. Expression data were used to identify *CamTERF* co-expression genes, which were then analyzed with goatools [52], which is a python package for GO enrichment analysis. 

### 4.8. ABA and Salt-Stress Treatment

A high-generation inbred line of capsicum, 6421, was used for ABA treatment experiments. After germination, the experimental plants were planted in trays and cultured with nutrient fluid in a climate-controlled chamber under a photoperiod of 16 h light/8 h dark and a day/night temperature of 27/18 °C. Considering the effects of circadian rhythm on gene expression, we did the untreated capsicum 6421 for experiments.

When there were four to six euphylla, ABA solution (30 μmol·L^−1^) was sprayed onto the whole plant. Leaf tissue was sampled at seven timepoints—0, 1, 1.5, 3, 6, 12, and 24 h after ABA treatment. Plants that were not treated with ABA, including seven timepoints, were used as control for eliminating the effects of light conditions. Three biological replicates, comprised of seven plants each, were included for each sampling time point. Next, samples were wrapped in aluminum foil and placed immediately into liquid nitrogen, then stored at −80 °C until further use. Salt stress experiments were conducted similar to the ABA treatment process, except that NaCl solution (200 mmol·L^−1^) solution was sprayed onto plants.

### 4.9. cDNA Generation and the Quantitative/Real-Time PCR Analysis

Total RNA was isolated from the four to six euphylla stages of capsicum 6421 using the RNAiso Plus reagent (TaKaRa, Dalian, China) and then treated with RNase-free DNase I (Promega). Subsequently, 0.5 μg RNA was used for first-strand cDNA synthesis using a HiScript II 1st Strand cDNA Synthesis kit (Vazyme, Nanjing, China) according to the manufacturer’s instructions. qPCR was performed using LightCycler 96 (Roche, Basel, Switzerland) with the SYBR Green Premix Ex Taq™ II quantitative PCR system (TaKaRa), and the primers of eight subclasses of genes are listed in Table S5. At least three biological replicates were included. Briefly, after an initial denaturation step at 95 °C for 10 min, the amplifications were carried out with 40 cycles at a melting temperature of 95 °C for 15 s and an annealing temperature of 60 °C for 30 s. The Δ*C*_t_ method was used to calculate the relative expression levels of *CamTERFs* [53,54]. Δ*C*_t_ = [Gene expression − mean (Actin expression)]/3.

## 5. Conclusions

This study analyzed capsicum *mTERFs* on a genome-wide level and identified 35 *CamTERFs*. Then, based on bioinformatic and RT-qPCR analyses, we characterized *CamTERF* gene structures, chromosomal locations, motif prediction, phylogeny, promoter cis-elements, and gene expression patterns in different tissues and in response to different abiotic treatments. A number of candidate *CamTERFs* were identified that may play important roles in ABA- and NaCl-stress responses. This research provides comprehensive information characterizing *CamTERFs*, which can help to identify *CamTERFs* functions.

## Figures and Tables

**Table 1:** The basic information of the mitochondrial transcription termination factor (*mTERF)* gene family in capsicum. List of predicted genes and related information include gene name (Zunla_1), CM334 homologous gene and E-value, gene locus, molecular details.

Gene	Gene Accession No.	CM334 Homelog	E-Value	Chr	Start	End	ORF bp	Size aa	Protein Molecular Weight/KD	PI
*CamTERF1*	*Capana00g003386*	*CA00g83930*	0	Chr00	544847272	544848822	1551	516	59.52	9.24
*CamTERF2*	*Capana00g003424*	*CA01g01340*	0	Chr00	545339784	545340731	948	315	35.86	9.3
*CamTERF3*	*Capana00g003922*	*CA04g12790*	1.25 × 10^−12^	Chr00	591637478	591637978	501	166	19.33	7.6
*CamTERF4*	*Capana01g003479*	*CA01g27850*	0	Chr01	227806286	227808022	1737	578	66.29	8.02
*CamTERF5*	*Capana01g003663*	*CA00g07130*	6.03 × 10^−41^	Chr01	232164334	232164957	624	207	24.25	9.13
*CamTERF6*	*Capana02g001023*	*CA02g09080*	0	Chr02	107229127	107230005	879	292	33.69	9.73
*CamTERF7*	*Capana02g001304*	*CA02g00270*	0	Chr02	119529666	119531393	1728	575	65.45	8.98
*CamTERF8*	*Capana02g002368*	*CA02g19520*	0	Chr02	143793081	143794292	1212	403	45.81	9.87
*CamTERF9*	*Capana02g003623*	*CA02g30630*	1.22 × 10^−17^	Chr02	163004467	163007189	1455	484	54.96	9.2
*CamTERF10*	*Capana03g000004*	*CA03g36450*	4.29 × 10^−69^	Chr03	95975	106725	1782	593	67.82	9.18
*CamTERF11*	*Capana03g001822*	*CA03g21280*	0	Chr03	36374556	36376173	1533	510	57.4	8.83
*CamTERF12*	*Capana03g003090*	*CA03g11980*	1.51 × 10^−17^	Chr03	168574645	168575497	729	242	28.26	9.22
*CamTERF13*	*Capana03g004299*	*CA03g04110*	0	Chr03	250309314	250310699	1386	461	53.08	9.25
*CamTERF14*	*Capana04g000706*	*CA04g17520*	0	Chr04	12169693	12170571	879	292	33.97	9.86
*CamTERF15*	*Capana04g001184*	*CA00g83430*	0	Chr04	36527349	36528682	1263	420	47.68	9.46
*CamTERF16*	*Capana04g001185*	*CA00g83420*	0	Chr04	36553095	36554216	1122	373	42.93	9.65
*CamTERF17*	*Capana04g001188*	*CA00g83410*	0	Chr04	36586260	36587354	1095	364	41.92	9.28
*CamTERF18*	*Capana04g001189*	*CA00g83400*	6.61 × 10^−18^	Chr04	36664744	36665883	1140	379	43.25	9.49
*CamTERF19*	*Capana04g001190*	*CA01g17910*	0	Chr04	36679829	36682422	1143	380	43.5	9.47
*CamTERF20*	*Capana04g001191*			Chr04	36685395	36685721	327	108	12.4	9.52
*CamTERF21*	*Capana04g001192*	*CA00g85840*	0	Chr04	36719507	36720607	1101	366	42.01	9.51
*CamTERF22*	*Capana04g001196*	*CA04g14910*	0	Chr04	36830156	36837562	1185	394	45.17	10.02
*CamTERF23*	*Capana04g001201*	*CA10g08900*	0	Chr04	36952799	36953611	813	270	30.41	9.42
*CamTERF24*	*Capana04g001203*	*CA00g85820*	0	Chr04	37032362	37033440	981	326	38	9.64
*CamTERF25*	*Capana04g001204*	*CA00g85830*	1.02 × 10^−15^	Chr04	37046910	37047527	618	205	23.63	9.52
*CamTERF26*	*Capana05g000328*	*CA05g03010*	7.52 × 10^−78^	Chr05	6905983	6907173	1191	396	45.16	9.67
*CamTERF27*	*Capana05g000329*	*CA05g03020*	0	Chr05	6908441	6909864	1173	390	44.77	10.02
*CamTERF28*	*Capana05g001366*	*CA05g09510*	0	Chr05	112851518	112852951	1434	477	53.95	6.1
*CamTERF29*	*Capana08g002535*	*CA00g64290*	0	Chr08	148385074	148388277	1971	656	75.9	9.16
*CamTERF30*	*Capana09g000680*	*CA09g07900*	0	Chr09	32649482	32650606	1125	374	43.07	9.65
*CamTERF31*	*Capana10g000180*	*CA10g01160*	0	Chr10	3405765	3407096	1332	443	50.49	6.12
*CamTERF32*	*Capana11g000966*	*CA11g10880*	0	Chr11	79029802	79030677	876	291	33.23	9.57
*CamTERF33*	*Capana11g001839*	*CA00g74110*	4.08 × 10^−15^	Chr11	197890867	197891505	639	212	24.48	8.93
*CamTERF34*	*Capana12g001016*	*CA08g18550*	0	Chr12	40961437	40962249	813	270	31.42	9.1
*CamTERF35*	*Capana12g001548*	*CA12g14250*	6.69 × 10^−15^	Chr12	119671476	119672487	930	309	35.48	9.55

**Table 2:** Subcellular localization of CamTERFs. Listed are the predicted protein output scores for chloroplast transit peptide (C), mitochondrial targeting peptide (M), signal peptide (S), or any other location (-). Location (Loc) details the subcellular localization predicted based on the prediction scores and reliability class (RC, 1–5) indicates the strength of the prediction, with 1 indicating the strongest prediction. RC is a measure of the size of the difference (‘diff’) between the highest (winning) and the second highest output scores.

Protein ID	Chloroplast Transit Peptide	Mitochondrial Targeting Peptide	Signal Peptide	Other Location	Loc	RC
CamTERF1	0.064	0.339	0.114	0.612	_	4
CamTERF2	0.888	0.047	0.016	0.07	C	1
CamTERF3	0.079	0.536	0.06	0.568	_	5
CamTERF4	0.062	0.883	0.008	0.21	M	2
CamTERF5	0.24	0.049	0.029	0.749	_	3
CamTERF6	0.098	0.127	0.165	0.532	_	4
CamTERF7	0.852	0.021	0.082	0.165	C	2
CamTERF8	0.049	0.681	0.007	0.028	M	2
CamTERF9	0.014	0.823	0.026	0.349	M	3
CamTERF10	0.862	0.067	0.01	0.138	C	2
CamTERF11	0.733	0.043	0.021	0.102	C	2
CamTERF12	0.018	0.567	0.125	0.16	M	3
CamTERF13	0.178	0.302	0.108	0.04	M	5
CamTERF14	0.106	0.27	0.019	0.732	_	3
CamTERF15	0.177	0.12	0.054	0.147	C	5
CamTERF16	0.082	0.857	0.006	0.031	M	2
CamTERF17	0.009	0.176	0.763	0.07	S	3
CamTERF18	0.014	0.797	0.163	0.042	M	2
CamTERF19	0.261	0.635	0.021	0.098	M	4
CamTERF20	0.06	0.164	0.142	0.883	_	2
CamTERF21	0.007	0.194	0.85	0.058	S	2
CamTERF22	0.086	0.773	0.008	0.082	M	2
CamTERF23	0.005	0.419	0.44	0.085	S	5
CamTERF24	0.274	0.078	0.024	0.39	_	5
CamTERF25	0.685	0.039	0.176	0.248	C	3
CamTERF26	0.069	0.284	0.085	0.06	M	5
CamTERF27	0.131	0.598	0.034	0.07	M	3
CamTERF28	0.212	0.659	0.026	0.029	M	3
CamTERF29	0.665	0.268	0.01	0.103	C	4
CamTERF30	0.009	0.538	0.611	0.025	S	5
CamTERF31	0.029	0.588	0.064	0.492	M	5
CamTERF32	0.071	0.339	0.012	0.707	_	4
CamTERF33	0.034	0.174	0.109	0.785	_	2
CamTERF34	0.815	0.029	0.203	0.017	C	2
CamTERF35	0.139	0.095	0.053	0.679	_	3

**Table 3:** List of putative motifs in *CamTERFs*.

Motif	Width	Sequence
1	21	YLVSHPALLMYSLEKRIKPRY
2	29	PKLLFYDVEKTLKPKLZFLKELGLSGSDL
3	50	VPPDSPMFLHGVQVLSSLKKSKLDRKJGIFKSFGWSDDDILTMFRKLPYC
4	31	YLRSLGGSDEBVVKLJKRCPWLLSYSLEKTL
5	21	YLINSLGFSKQEAJSASAKVT
6	22	SESKFLEKYVLPYKDELPDLYE
7	21	SEARIQTALTFFMKELGYKSA
8	28	LLRNFGFSNDKIRKMVLRCPQLLTQNPE
9	21	LVVNFFKQTGFBBTQIKKLVS
10	21	VKVIARDPKLLTRSLDTHJKP

## Supplementary Materials

Supplementary materials can be found at https://www.mdpi.com/1422-0067/21/1/269/s1.

## Abbreviations

mTERFs	Mitochondrial transcription ter
CamTERFs	Capsicum annuum Mitochondr
FPKM	Fragments Per Kilobase Million
GO	Gene Ontology
MeJA	Methyl Jasmonate
BP	Biological Process
MF	Molecular Function
CC	Cellular Component
RNA-Seq	RNA sequencing;
mtDNA	Mitochondrial DNA
ABA	Abscisic acid
RT-qPCR	Reverse transcription quantitative PCR
